# Effects of early feeding of enzymatically treated yeast on growth performance, organ weights, intestinal histomorphology, and ceca microbial metabolites in broiler chickens subjected to *Eimeria* challenge

**DOI:** 10.1016/j.psj.2022.101967

**Published:** 2022-05-21

**Authors:** Elijah G. Kiarie, Mohsen Mohammadigheisar, Hagen Schulze

**Affiliations:** ⁎ Department of Animal Biosciences, University of Guelph, Guelph, ON N1G 2W1, Canada; ϯ Livalta, AB Agri Ltd., Peterborough, Cambridgeshire, PE2 6FL, UK

**Keywords:** broiler chickens, *Eimeria*, enzymatically treated yeast, growth performance, physiological responses

## Abstract

The study evaluated effects of early feeding of enzymatically treated yeast on growth performance and selected physiological responses in broiler chickens. A total of 480-day-old (male) Ross × Ross 708 broiler chicks were placed in 24 floor pens (20 birds per pen) and allocated to 2 diets (control vs. yeast) in a completely randomized block design (n = 12). Diets were formulated for a 5-phase feeding program: Pre-starter; d 0 to 6 Starter; d 7 to 15, Grower: d 16 to 28, Finisher 1; d 28 to 42 and Finisher 2; d 43 to 56. The yeast was applied in pre-starter and starter diets at 0.6 and 0.2%, respectively. Birds received a common diet from d 16 to 56. Feed intake (**FI**) and body weight (**BW**) were recorded by phase for calculation of BW gain (**BWG**) and FCR. On d 10, all birds received an oral dose of 25,000 *E. acervullina* and 5,000 *E. maxima* sporulated oocysts in 1 mL of sterile saline. On d 15 post-hatch, one bird per pen was sacrificed for organ weights (gizzard, small intestine, ceca, liver, spleen, liver, and bursa), jejunal tissues for histomorphology and ceca digesta for microbial activity. On d 56, one bird per pen was sacrificed for organs and breast weight. In pre-starter phase, yeast fed birds showed improved (*P* < 0.05) BWG and FCR than control fed birds. Combining pre-starter and starter phases, the FCR of yeast fed birds showed improved FCR (1.115 vs. 1.135; *P* < 0.05) than control. The overall BWG (d 0–56) was 3.920 and 3.962 kg/ bird and corresponding values for FCR were, 1.808 and 1.755, for the control and yeast, respectively. Diets had no (*P* > 0.05) effects on physiological responses evaluated on necropsied birds except that yeast birds had (*P* < 0.05) lighter bursa than control birds on d 15. The current data indicated that yeast could support growth in early life of broiler chickens, but these effects were not sustained after the transitioning birds to common grower and finisher diets.

## INTRODUCTION

There is increasing consumer demand for birds raised on feeding programs without or reduced antimicrobial growth promoters (**AGP**) in conjunction with evolving specialty production practices such as organic, all vegetable and pasture feeding regimens (Bean-Hodgins and Kiarie, 2021). In cognizant of the critical role of establishing healthy and functional gastrointestinal in early life and subsequent impact on long-term performance, there is tremendous ongoing industry and academia research effort. Much efforts have been dedicated on the development of specialized starter feeding programs based on a range of digestible and functional ingredients such as epidermal growth factor, specialty soy products, advanced feed processing among others (Douglas et al., 2014; Barekatain and Swick, 2016; Kim et al., 2017; Kim et al., 2018, 2020; Kiarie and Mills, 2019; Kiarie et al., 2021). Further efforts have been dedicated on specialty feed additives as alternatives to AGP (Kiarie et al., 2013; Kiarie et al., 2016; Kiarie et al., 2019).

Studies have examined the use of yeast and yeast derivatives, including live, inactivated, cultured, autolyzed, cell wall, or content extracted or isolated products, as natural growth and performance enhancing supplements for poultry (Ahiwe et al., 2019). *Saccharomyces cerevisiae* is a highly adaptable organism with significant industrial importance for example ethanol, bread, single cell protein and vitamin production (Kurtzman et al., 2011; Jansen et al., 2017). Of particular interest in yeast are the functional components of cell contents such as peptides, enzymes, nucleotides, and cell wall constituents such as β-glucans, glycoproteins, mannans, and chitin (Kollár et al., 1997; Cabib et al., 2008). Indeed, the growth-enhancing and immune-modulatory potentials of yeast products in poultry production have been attributed to the presence of α-mannan and β-glucans components (Ahiwe et al., 2019).

Production of specialty products is seen as a key differentiator of many yeast-based functional feed additives available to the poultry industry. As heterotrophic organisms, energy and carbon metabolism are intimately interconnected giving yeast cells ability to produce wide variety of derivatives depending on the composition of the fermentation media and the fermentation conditions (Hatoum et al., 2012). It follows that yeast culture production can be manipulated to produce unique feed additives that contain single or combination of derivatives beneficial to animal nutrition and health. The individual components have differing modes of action in the gut and can be valuable in maintaining performance when the use of therapeutic and prophylactic compounds is reduced or restricted (Shurson, 2018; Kiarie et al., 2019). *Saccharomyces cerevisiae* derived from the sugarcane-based bio-ethanol fermentation is characterized to contain exceptionally high levels of β-glucans and mannan oligosaccharides, a pre-requisite for described yeast cell wall functionalities such as bacteria binding and modulation of the immune system. These attributes are further enhanced though enzymatic treatment of cell walls (Lu et al., 2019). We aimed to investigate whether application of this yeast in pre-starter and starter diets (≤15 d of age) was beneficial in the development of a healthy gastrointestinal tract to positively impact growth performance of broiler chickens subjected to *Eimeria* challenge through to slaughter weight.

## MATERIALS AND METHODS

Animal care and use protocols were approved by the University of Guelph Animal Care and Use Committee and birds were cared for in accordance with the Canadian Council on Animal Care guidelines (CCAC, 2009).

### Test Ingredients and Dietary Treatments

The yeast was an enzymatically treated non-GMO *Saccharomyces cerevisiae* containing 40% cell wall components (β-1-3 and 1-6 glucans and mannan oligosaccharides) and 36% crude protein (Livalta Cell HY40, Livalta, Ab Agri Ltd., Cambridgeshire, Peterborough, UK). The typical analyses (% as fed) of the tested yeast were 92, 28, 12, 10, 1 for dry matter, total β-glucans, total mannan, crude ash and crude fiber, respectively. The concentration of amino acids (% CP as fed) was 7.30, 1.61, 0.65, 5.11, 4.86, 3.88, and 5.67 for lysine, methionine, cysteine, threonine, isoleucine, arginine, and valine, respectively. The diets were formulated for a 5-phase feeding program: pre-starter; d 0 to 6 starter; d 7 to 15, grower: d 16 to 28, finisher 1; d 28 to 42, and finisher 2; d 43 to 56, to meet or exceed the nutrient requirements of Ross × Ross 708 recommended by the breeder (Aviagen, 2014) with exception of reduction of SID Lys and SID Met content by 3% and SID Thr content by 10% in each phase (Table 1). The yeast was supplemented in pre-starter (0.6%) and starter (0.2%) phases only creating a control and yeast supplemented diets for these phases. The yeast was dosed based on supplier recommendations and was included by replacing a small amount of corn. Differences in yeast inclusion in pre-starter and starter phases are to account for expected increase in feed intake as bird aged. The birds received a common diet from grower to the end of the experiment. All diets contained 500 FTU of phytase /kg (Quantum Blue, AB Vista, Marlborough, UK) and were free of anticoccidial or antimicrobial growth promoting or alternatives and animal by-products (pork meal, poultry by-products etc.). The pre-starter and starter were prepared in fine crumble form, grower feed was in coarse crumble form, and finisher was prepared in short pellet form. The temperature of the processing condition was 60 to 65˚C and steam pressure of 30 psi. Samples of feed were collected for nutrient analyses.

### Birds and Housing

A total of 480-day-old (male) Ross × Ross 708 broiler chicks were procured from a commercial hatchery (Maple Leaf Foods, New Hamburg, ON, Canada), weighed and allocated to 24 floor pens (20 birds per pen) based on BW. The pens were housed in environmentally controlled rooms with 12 pens each (each pen provides 46 sq ft area) and bedded with fresh wood shavings. The room temperature was set to breeder recommendation of 32°C on d 0 and gradually decreased to 27°C by d 17. Birds were exposed to fluorescent lighting in a 23 h of light (20+ lux) for the first 4 d and then a 16 light: 8 dark (10–15 lux) light cycle for the remainder of the experiment in accord with Arkell Poultry Research Station standard operating procedures.

### Experimental Procedures, Measurements, and Sampling

The diets were allocated to pens in a completely randomized complete block (room) design to give 12 replicates per treatment. Birds had free access to water via nipple drinkers and feed via feeders throughout the experiment. Body weight and feed intake was monitored at on d 0, 6, 15, 28, 42, and 56 for calculation of body weight gain (**BWG**) and feed conversion ratio (**FCR**). Mortalities were counted, and BW recorded for adjusting FCR. On d 10 post-hatch, all birds were challenged with 25,000 *E. acervullina* and 5,000 *E. maxima* sporulated oocysts suspended in 1 mL saline solution (Akbari Moghaddam Kakhki et al., 2019; Leung et al., 2019a,b). The intent of *Eimeria* challenge was to subject birds in farm-like conditions, we have demonstrated this model was effective in causing consistent lesions in the small intestines (Kiarie et al., 2019). On d 15 and 56 post-hatch, 1 bird/pen was randomly selected, weighed, and euthanized via cervical dislocation. Weights of empty gizzard, small intestine, and ceca, along with liver, spleen, and bursa were recorded. For d 56 birds, breast was dissected and weighed. Jejunal segments (∼3 cm) were excised and placed in buffered formalin for histomorphology analysis. For d 15 birds, ceca digesta samples (1 bird per pen) were collected using BioFreeze sampling kits following the recommended protocol by the manufacturer and shipped to Alimetrics laboratories Ltd (Espoo, Finland) for processing and analyses of concentration of total bacteria and short chain fatty acids (**SCFA**).

### Laboratory Analyses

Diet samples were finely ground and submitted to a commercial lab (SGS Canada, Guelph) for dry matter, crude protein, crude fat, starch, ethanol soluble carbohydrates, and minerals analyses. Fixed jejunal tissues were cut into a longitudinal cross section and embedded in paraffin wax. The tissues were then sectioned (5 µm) and stained with haematoxylin and eosin for morphological measurements. A total of 5 villus-crypt structures were measured with a calibrated micrometre for each tissue using a Leica DMR microscope (Leica Microsystems, Wetzlay, Germany). Villus height and crypt depth ratio (**VH:CD**) were calculated. Total bacteria in ceca digesta was determined using quantitative real-time polymerase chain reaction (**qPCR**) method as we recently described (Kiarie et al., 2021). Briefly, the samples were washed with sterile physiological saline solution to remove solid particles and complex polysaccharides to improve subsequent DNA purification and the downstream qPCR applications. The liquid phase was subjected to differential centrifugation for collecting the bacterial cells. The microbial cell walls were disrupted, and the chromosomal DNA was quantitatively extracted and quantified using a Nanodrop 2000 spectrophotometer (ThermoScientific, Wilmington, DE). The total bacteria were determined based on detection and quantification of a fluorescent reporter signal that increases in direct proportion to the amount of PCR product in the reaction. The primer for the target microbiota assessed in present study was previously reported (Apajalahti et al., 2007; Kettunen et al., 2017; Apajalahti et al., 2019). The data was reported as number of copies of 16S RNA per gram of sample. The SCFA in ceca digesta was derivatized to the respective phenyl esters by using phenyl chloroformate reagent and analyzed using gas chromatography (Agilent Technologies, Santa Clara, CA) with pivalic acid (Sigma-Aldrich, St. Louis, MO) as an internal standard. The chromatography procedure which used a glass column packed with 80/120 Carbopack B-DA/4% Carbowax stationary phase, helium as a carrier gas, and a flame ionization detector has been described previously by Apajalahti et al. (2019).

### Statistical Analyses

Data were evaluated for the presence of outliers using box and whisker method and subsequently subjected to statistical analyses using PROC MIXED procedures of SAS with pen as the experimental unit. The model had diet as fixed and block as random effects. Significance was declared at *P* < 0.05 and least square means separated by student *t* test.

## RESULTS

Mortalities were very low in the present study. Specifically, the total mortalities for control and yeast diets, respectively were 2 and 3 birds in pre-starter, 1 and 2 birds in grower, 2 and 0 birds in finisher 1, and 5 and 3 birds in finisher 2. Table 2 shows analyzed chemical composition of experimental (pre-starter and starter) and common (grower, finisher) diets. The birds fed yeast were heavier by 10 g on d 6 post-hatch, improved BWG (*P* < 0.01) and FCR (*P* < 0.01; Table 3). Although there was no diet effect (*P* > 0.05) on BWG, FI and FCR in starter phase, birds fed yeast showed improved FCR (1.115 vs. 1.135; *P* = 0.008) when pre-starter and starter phases were combined. Feeding yeast in pre-starter and starter phase did not influence growth performance when birds were transitioned in common diets (grower and finisher phases) and in the overall (d 0–56). Gizzard, small intestine, ceca, and liver weight were not (*P* > 0.05) influenced by diets (Table 4). Birds fed yeast had tendency (*P* = 0.059) for lighter absolute bursa than control birds on d 15. However, birds fed yeast had lower bursa index (0.187 vs. 0.213 g/g BW; *P* = 0.049) than birds fed control diet. There were no (*P* > 0.05) diet effects on bursa weight of index on d 56. However, birds fed yeast had numerically higher (∼+23%) bursa and bursa index than control birds. Diets had no (*P* > 0.05) effects on spleen attributes at any age. Diets had no effects on jejunal villus height, crypt depth, and their ratios (Table 4). The concentrations of total bacteria and SCFA were not affected by diets (Table 5).

## DISCUSSION

The modern broiler increases its body weight by 25% overnight and 5,000% by 5 wk of age (Aviagen, 2019). Additional weight achieved by d 7 is compounded by subsequent weekly percentage increases translating into greater weight for age. For example, every extra gram of body weight achieved at d 7 could result in an extra 5 g of body weight at d 49 (Leeson, 2008). In essence a chick at 190 vs*.* 150 g at 7 d can be expected to be 200 g heavier at 49 d. Most vital organs reach their maximum relative functions during the first week of life. Thus, provision of proper nutrition at an early age will affect life-long productivity. Yet feed consumption is low and highly variable and during this period, presenting a challenge for delivery of critical nutrients for growth. Moreover, because of immature digestive and immune systems, the newly hatched chicks are highly susceptible production environment stressors. Therefore, formulating pre-starter and starter diets revolves around the selection of digestible and functional ingredients in alignment with immature digestive capacity.

We investigated utility of enzymatically treated whole non-GMO *Saccharomyces cerevisiae* strain rich in β-1.3/1.6 glucans and mannan oligosaccharides in broiler chicken. The birds were subjected to *Eimeria* challenge to create mimicry farm conditions. *Eimeria* infection is associated with reduction in digestion, absorption linked to morphological and functional intestinal damage (Kim et al., 2017; Leung et al., 2019a,b). The ensuing mucogenesis and enterocyte turnover, and immune system activation have negative effects on nutrients utilization (Williams, 2005). Yeast cell and cell wall components have been demonstrated to modulate cellular and humoral mediated immune responses against coccidia infections (Elaine-Rose and Long, 2009; Leung et al., 2019a,b). In this context we hypothesized that birds fed yeast will have advantage over the control. Supplementation of yeast improved growth and FCR in pre-starter and FCR in starter phase, however, these effects were not apparent when birds were transition to common diets. Although yeast inclusion was 0.6 and 0.2% for pre-starter and starter phases, respectively, birds consumed remarkably 0.762 gram of yeast in either of the 2 phases based on observed feed intake. This validated our inclusion strategy on account of expected feed intake in pre-starter and starter phases. Perhaps suggesting the need for continuous feeding to sustain the benefits. A meta-analyses of published research on *Saccharomyces cerevisiae var. boulardii* cell walls supplementation in broiler chicken diets revealed improvement on growth performance over control (Hooge, 2004). A study that supplemented isolated yeast β-1,3/1,6-glucan at various doses 0, 25, 50, 75, 100, and 125 mg/kg) supplementation throughout the entire experiment (d 0–42) observed a quadratic increase in growth performance (Zhang et al., 2008).

Lymphoid organs are an appropriate target for determining modulation of immune competency in poultry (Cazaban et al., 2015). The bursa of Fabricius is considered the primary lymphoid organ in poultry and is critical in differentiation of B-lymphocytes (Schat and Skinner, 2014). In general yeast supplementation is expected to stimulate enlargement of lymphoid organs (Zhang et al., 2008). However, we observed an opposite effect in the present study. The reason for this observation is not clear. However, bursa index responses to supplementation of yeast and yeast metabolites have been variable. For example, Zhang et al. (2008), fed broiler chickens increments of 25 up to 125 mg yeast β-1,3/1,6-glucans/kg of feed and observed a quadratic response on bursa indices of broiler chickens. On d 21, birds fed 50 and 75 had higher bursa indices than birds fed control while other treatments were similar to control. However, on d 42, yeast β-1,3/1,6-glucans linearly increased bursa indices to 100 mg/kg supplementation. Although we did not observe yeast effects on bursa attribute on d 56, it was notable a numerically heavier bursa was apparent in birds fed yeast. Leung et al. (2019a), reported feeding yeast product increased bursa weight in 35 and not in 15-day-old broiler chickens. This may indicate yeast metabolites induced enlarged lymphoid organs later in broiler life (Cooper et al., 1966). An increase in bursa in yeast nucleotides fed broilers was associated with increased IgA production boost mucosal immunity (Daneshmand et al., 2017). Nucleotides are among the components in hydrolyzed whole yeast cells. Feeding broilers enzymatically treated yeast cell had no effects on bursa weight in 9-day-old broiler chickens (Lu et al., 2019). Gastrointestinal microbiota activity influences immunity, nutrient absorption, and growth performance in birds. Unlike the present study, yeast derivatives have been demonstrated to improve intestinal histomorphology and modulate ceca microbial activity in other studies (as reviewed Kiarie et al., 2019).

Because of immature digestive and immune systems, smooth transition of newly hatched chicks to production environment has huge impact on lifetime biological and economic performance. Thus, strategies that bolster growth in early phase of chick life have practical implications on optimal broiler chicken production. In the current study yeast supported growth in early life of broiler chickens. However, these effects were not sustained after the transition of the birds to common grower and finisher diets despite the birds being subjected to *Eimeria* challenge. Future studies should explore evaluation of the yeast supplementation throughout production period. Moreover, studies in dose response of yeast supplementation are also warranted.

**Table 1:** Ingredient and nutrient composition of experimental diets, as fed basis.

Item	Pre-starter (d 0–6)		Starter (d 7–15)		Grower (d 16–28)	Finisher 1 (d 29–42)	Finisher 2 (d 43–56)
	Control	Yeast	Control	Yeast			
Ingredients, %							
Corn	41.6	41.1	43.3	43.1	46.6	51.3	56.1
Soybean meal	42.5	42.2	40.5	40.4	36.9	32.0	27.7
Wheat	7.00	7.00	7.00	7.00	7.00	7.00	7.00
Limestone	1.41	1.41	1.33	1.33	1.22	1.11	1.06
Sodium chloride	0.34	0.34	0.35	0.35	0.34	0.34	0.33
Monocalcium phosphate	0.86	0.86	0.77	0.77	0.64	0.53	0.47
Sodium bicarbonate	0.19	0.19	0.18	0.18	0.19	0.16	0.20
L -Lysine HCl	0.16	0.15	0.13	0.13	0.15	0.15	0.18
DL -Methionine	0.32	0.32	0.29	0.29	0.29	0.27	0.24
L -Threonine	0.07	0.06	0.05	0.04	0.10	0.09	0.09
Vitamin-trace mineral premix^1^	1.00	1.00	1.00	1.00	1.00	1.00	1.00
Phytase^2^	0.01	0.01	0.01	0.01	0.01	0.01	0.01
Soybean oil	4.51	4.81	5.12	5.22	5.58	6.00	5.57
Yeast^3^	0.00	0.60	0.00	0.20	0.00	0.00	0.00
Calculated composition							
Crude protein, %	23.0	23.0	22.2	22.2	20.9	19.1	17.6
AME, mcal/kg	3.00	3.00	3.06	3.06	3.13	3.21	3.23
SID Lys, %	1.24	1.24	1.17	1.17	1.11	1.01	0.94
SID Met + Cys, %	0.92	0.92	0.88	0.88	0.85	0.79	0.73
SID Thr, %	0.77	0.77	0.73	0.73	0.74	0.67	0.63
Ca, %	0.96	0.96	0.91	0.91	0.84	0.77	0.73
Available P, %	0.48	0.48	0.46	0.46	0.42	0.39	0.36
Na, %	0.18	0.18	0.18	0.18	0.18	0.17	0.18
Cl, %	0.23	0.23	0.23	0.23	0.23	0.23	0.23
DEB, mEq/kg	283	285	273	273	257	230	215

1 Provided per kilogram of diet: vitamin A, 8,800.0 IU; vitamin D_3_, 3,300.0 IU; vitamin E, 40.0 IU; vitamin B_12_, 12.0 mg; vitamin K_3_, 3.3 mg; niacin, 50.0 mg; choline, 1,200.0 mg; folic acid, 1.0 mg; biotin, 0.22 mg; pyridoxine, 3.3 mg; thiamine, 4.0 mg; calcium pantothenic acid, 15.0 mg; riboflavin, 8.0 mg; manganese, 70.0 mg; zinc, 70.0 mg; iron, 60.0 mg; iodine, 1.0 mg; copper, 10 mg; and selenium, 0.3 mg.

2 Provided 500 FTU of phytase per kg of feed supplying provided 0.15% available P and 0.16% Ca (Quantum Blue, AB Vista, Marlborough, UK).

3 Enzymatically treated whole non-GMO *Saccharomyces cerevisiae* strain, 40% cell wall components (β-1.3/1.6 glucans and mannan oligosaccharides) and 36% crude protein (Livalta TMCell HY40, AB AGRI, Peterborough, UK). Dietary electrolyte balance, calculated as follows [(Na × 434.78) + (K × 255.75) − (Cl × 281.69)].

**Table 2:** Analyzed chemical composition of experimental diets, as fed.

Item	Pre-starter (d 0–6)		Starter (d 7–15)		Grower (d 16–28)	Finisher 1 (d 29–42)	Finisher 2 (d 43–56)
	Control	Yeast	Control	Yeast			
Dry matter, %	89.1	89.2	87.6	87.6	88.6	87.3	87.7
Crude protein, %	25.5	26.0	23.6	23.5	23.3	21.2	19.6
Crude fat, %	5.90	6.14	6.28	7.44	8.25	8.46	8.10
Starch, %	32.9	31.3	31.3	31.1	32.9	35.7	37.7
ESC^1^, %	4.57	5.22	4.05	6.82	4.74	4.74	4.71
Calcium, %	1.00	0.84	0.75	0.68	0.61	0.19	0.58
Phosphorous, %	0.62	0.61	0.57	0.58	0.55	0.50	0.48
Potassium, %	1.08	1.16	1.09	1.05	1.02	0.94	0.88
Magnesium, %	0.19	0.20	0.18	0.18	0.18	0.16	0.16
Sodium, %	0.18	0.17	0.19	0.21	0.21	0.21	0.19

1 Ethanol soluble carbohydrates (simple sugars).

**Table 3:** Effects of yeast supplementation on growth performance in broiler chickens, d 0–56.

Item	Pre-starter	Starter	Grower	Finisher-1	Finisher-2	Yeast diet	Common diet	Overall
	(d 0–6)	(d 7–15)	(d 16–28)	(d 29–42)	(d 43–56)	(d 0–15)	(d 16–56)	(d 0–56)
Body weight, kg/bird								
Control	0.169^b^	0.492	1.517	2.860	4.045	-	-	-
Yeast	0.179^a^	0.497	1.523	2.863	4.051	-	-	-
SEM^1^	0.002	0.003	0.013	0.028	0.056	-	-	-
*P*-value	<0.01	0.384	0.757	0.932	0.940			
Body weight gain, kg/bird								
Control	0.128^b^	0.323	1.022	1.329	1.117	0.451	3.468	3.920
Yeast	0.137^a^	0.318	1.022	1.340	1.145	0.455	3.507	3.962
SEM^1^	0.001	0.003	0.014	0.022	0.060	0.003	0.060	0.060
*P*-value	<0.01	0.141	0.964	0.724	0.742	0.485	0.651	0.622
Feed intake, kg/bird								
Control	0.127	0.386	1.368	2.310	2.876	0.513	6.553	7.067
Yeast	0.127	0.381	1.376	2.308	2.757	0.508	6.441	6.949
SEM^1^	0.001	0.003	0.012	0.022	0.066	0.003	0.083	0.084
*P*-value	0.836	0.211	0.636	0.945	0.217	0.328	0.346	0.334
Feed conversion ratio								
Control	0.992^a^	1.193	1.339	1.742	2.686	1.136^a^	1.897	1.808
Yeast	0.928^b^	1.198	1.348	1.724	2.440	1.117^b^	1.838	1.755
SEM^1^	0.007	0.006	0.010	0.020	0.145	0.005	0.031	0.027
*P*-value	<0.01	0.560	0.538	0.535	0.245	0.008	0.197	0.174

1 SEM standard error of means. Data are least squares means of 12 replicate pens/treatment.

abc Values with different superscripts for a parameter differ (*P* < 0.05).

**Table 4:** Effects of yeast supplementation on visceral organs weight, lymphoid organs attributes and jejunal histomorphology in broiler chickens.

	D 15				D 56			
Item	Control	Yeast	SEM	*P*-value	Control	Yeast	SEM	*P*-value
Organ weight, g/kg BW								
Gizzard	27.1	28.1	1.054	0.506	13.3	15.1	0.775	0.120
Small Intestine	54.4	51.3	1.472	0.140	20.7	19.0	0.716	0.128
Liver	30.8	29.0	0.774	0.072	17.0	17.3	0.577	0.710
Ceca	4.97	4.79	0.226	0.576	2.94	2.93	0.145	0.964
Lymphoid organs attributes								
Bursa weight, g	1.04	0.91	0.044	0.059	5.29	6.51	0.602	0.168
Bursa index, g/g BW × 100	0.213^a^	0.187^b^	0.008	0.049	0.128	0.157	0.014	0.153
Spleen Weight	0.506	0.528	0.032	0.641	4.80	4.98	0.357	0.720
Bursa index, g/g BW × 100	0.104	0.108	0.006	0.651	0.116	0.120	0.008	0.695
Breast weight	-	-	-	-	285	278	6.959	0.472
Jejunal histomorphology								
Villus height (VH), µm	1,229	1,134	59.5	0.278	1,300	1,179	72.575	0.269
Crypt depth (CD), µm	264	259	18.5	0.869	185	189	18.928	0.866
VH:CD ratio	4.97	4.62	0.419	0.569	7.56	6.58	0.536	0.197

Data are least squares means of 12 replicate pens/treatment.

**Table 5:** Effects of yeast supplementation on concentration of total bacteria and short chain fatty acids (SCFA) in ceca digesta of 15-day-old broiler chickens.

Item	Control	Yeast	SEM	*P*-value
Log_10_ total eubacteria, 16S genes/g	12.28	12.34	0.058	0.519
SCFA concentration, mmol/kg				
Acetic acid,	71.20	77.02	7.858	0.606
Propionic acid	3.17	4.42	0.765	0.264
Butyric acid	14.81	18.24	2.273	0.309
Valeric acid	1.26	1.69	0.204	0.169
Lactic acid	3.34	3.51	1.626	0.950
Total SCFA^1^	92.88	101.47	10.356	0.564
SCFA molar proportion, %				
Acetic acid,	76.79	77.57	2.180	0.803
Propionic acid	3.68	4.53	0.763	0.440
Butyric acid	15.46	16.18	1.417	0.728
Valeric acid	1.42	1.63	0.207	0.487
Lactic acid	3.64	3.12	1.345	0.805

Data are least squares means of 12 replicate pens/treatment.

1 Summation of acetic, propionic, butyric, valeric and lactic acids.
